# Amino Acid Profiles and Biopotentiality of Hydrolysates Obtained from Comb Penshell (*Atrina pectinata*) Viscera Using Subcritical Water Hydrolysis

**DOI:** 10.3390/md19030137

**Published:** 2021-03-01

**Authors:** Hee-Jeong Lee, Vikash Chandra Roy, Truc Cong Ho, Jin-Seok Park, Yu-Rin Jeong, Seung-Chan Lee, Sung-Yeol Kim, Byung-Soo Chun

**Affiliations:** 1 Institute of Food Science, Pukyong National University, 45 Yongso-ro, Nam-gu, Busan 48513, Korea; leehjeong@ks.ac.kr (H.-J.L.); hocongtruc@pukyong.ac.kr (T.C.H.); seungchan229@pukyong.ac.kr (S.-C.L.); 2 Department of Food and Nutrition, Kyungsung University, 309 Suyeong-ro, Nam-gu, Busan 48434, Korea; 3 Department of Food Science and Technology, Pukyong National University, 45 Yongso-ro, Nam-gu, Busan 48513, Korea; vikashft@hstu.ac.bd (V.C.R.); jin1931@pukyong.ac.kr (J.-S.P.); jeongyurin@pukyong.ac.kr (Y.-R.J.); kim41409114@pukyong.ac.kr (S.-Y.K.); 4 Department of Fisheries Technology, Hajee Mohammad Danesh Science and Technology University, Dinajpur 5200, Bangladesh

**Keywords:** *Atrina pectinata*, subcritical water hydrolysis, amino acid profile, SDS-PAGE, antioxidant activity, anticoagulant activity, antihypertensive activity

## Abstract

The recovery of amino acids and other important bioactive compounds from the comb penshell (*Atrina pectinata*) using subcritical water hydrolysis was performed. A wide range of extraction temperatures from 140 to 290 °C was used to evaluate the release of proteins and amino acids. The amount of crude protein was the highest (36.14 ± 1.39 mg bovine serum albumin/g) at 200 °C, whereas a further increase in temperature showed the degradation of the crude protein content. The highest amount of amino acids (74.80 mg/g) was at 230 °C, indicating that the temperature range of 170–230 °C is suitable for the extraction of protein-rich compounds using subcritical water hydrolysis. Molecular weights of the peptides obtained from comb penshell viscera decreased with the increasing temperature. SDS-PAGE revealed that the molecular weight of peptides present in the hydrolysates above the 200 °C extraction temperature was ≤ 1000 Da. Radical scavenging activities were analyzed to evaluate the antioxidant activities of the hydrolysates. *A. pectinata* hydrolysates also showed a particularly good antihypertensive activity, proving that this raw material can be an effective source of amino acids and marine bioactive peptides.

## 1. Introduction

The comb penshell, *Atrina pectinata,* is a marine bivalve belonging to the phylum Mollusca, and is found mostly in subtidal areas of the Indian and Western Pacific Oceans [1,2,3]. South Korea has reported five species of the comb penshell; among these, thousands of tons of *A. pectinata* are harvested every year [4]. The adductor muscle of this large bivalve is a popular type of seafood in East Asian countries [5]; it has large amounts of amino acids, polyunsaturated fatty acids, and different health-beneficial minerals [6]. A large amount of remaining parts, such as the viscera (approximately 25% of the total body weight), are generated as waste in bivalve-processing factories [5]. Shellfish viscera are a proven rich source of protein, containing various bioactive peptides that have antioxidant, anti-inflammatory, antithrombotic, and other biological activities [7,8]. From the consumption pattern of the comb penshell, of which only its muscle is largely eaten, the remaining viscera and shell can be a valuable source of obtaining different biopolymers. Crustacean shells are an excellent source of different bioactive compounds (carotenoids) [9,10], biopolymers (chitin and chitosan), and minerals [11].

Subcritical water is a state of water in the subcritical zone, where the temperature ranges from 100 to 374 °C and the pressure is enough to keep the water in its liquid state; this water is also called “hot compressed water”, “hydrothermal water”, or “superheated water” [12]. Subcritical water extraction is a safe and green extraction technology, where the high ionization constant leads to more H^+^ and OH^−^ ions generated from water; this is similar to an extraction process of acids/alkalis of various bioactive compounds, such as peptides [13]. The extraction of protein from raw material and its conversion to low-molecular-weight peptides using this green technology are gaining popularity nowadays, instead of chemical and enzymatic processes [14]. The application of enzymes to hydrolyze proteins is time consuming, requires acids and bases to maintain the extraction condition, and is an expensive method. On the other hand, strong acids or bases are required for the chemical conversion of protein to peptides, which have a negative impact on the environment. Moreover, peptides obtained from chemical conversion contain a large amount of salt, as a result of the pH neutralization [15]. However, green technology, such as subcritical water hydrolysis (SWH), can be the best alternative to obtain good-quality peptides, as it is environmentally friendly, cost-effective, efficient, and has faster extraction efficiency. Recovering peptides and amino acids from fishery by-products using environmentally friendly processes will help the conversion of low-grade raw materials into various bioactive compounds, such as proteins and peptides, that have direct applications in the food, pharmaceutical, and cosmetic industries [16].

The extraction temperature is among the crucial parameters for subcritical hydrolysis, which significantly influences the hydrolysis of proteins [13]. It also influences the molecular size of the peptides and the production of amino acids [15]. Several studies have been conducted to obtain different temperature-induced hydrolysates from bivalve viscera, focusing on several physicochemical activities, such as the total protein content, color, pH, and antioxidant activities. However, the subcritical water-assisted extraction of peptides and amino acids from marine bivalves has rarely been studied.

Hence, the present study focused on the preparation of amino acid-rich visceral hydrolysate obtained from *A. pectinata* using SWH. The physicochemical and antioxidant activities of the hydrolysates prepared at different temperature ranges were also analyzed. Amino acid analyses and biological activities of the hydrolysates were analyzed at a temperature of 140–290 °C at the reaction time of 15 min. As far as we know, there is no prior study on the utilization of comb penshell viscera; therefore, we believe that this research work will generate a new idea to produce crustacean visceral hydrolysates and the subsequent preparation of amino acids and peptides of high commercial value in the food, pharmaceutical, and cosmetic industries.

## 2. Results and Discussion

### 2.1. Proximate Composition

The proximate composition of lyophilized *A. pectinata* powder is presented in Table S1. The raw material showed 60.70% ± 0.11% crude protein, which indicates that it was suitable for the extraction of peptides and amino acids. Other components, namely, crude lipids, ash, and carbohydrates, were 9.60% ± 0.13%, 17.20% ± 0.05%, and 9.98% ± 0.05%, respectively. The raw material was completely freeze-dried, with only 2.50% ± 0.02% moisture.

### 2.2. Hydrolysis Efficiency

**Figure 1:**
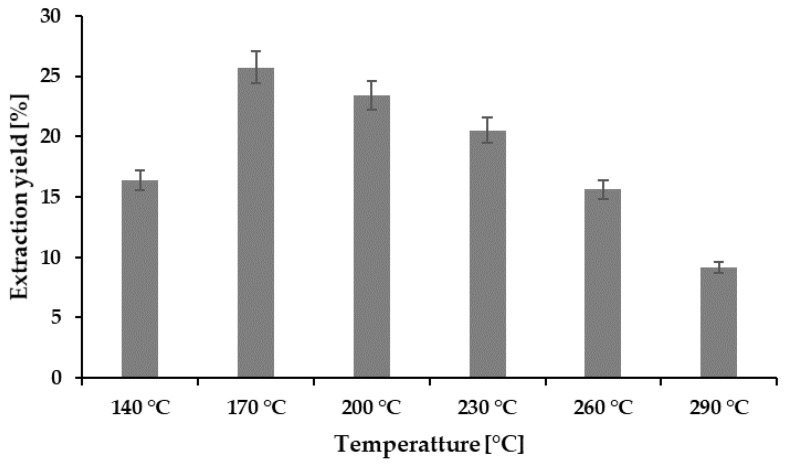
Extraction yield (%) at different temperatures.

The extraction yield obtained at various temperatures was converted to the HE presented in Figure 1. Among the hydrolysates obtained, the highest extraction yield of 25.74% was obtained at 170 °C, but the yield showed a decreasing pattern with the increase in the extraction temperature. A similar pattern has also been reported by the recently published [7] subcritical studies of abalone viscera. The HE is largely dependent on the dissolution capacity of the subcritical water, which is related to the temperature and pressure [17]. Although it is claimed that the temperature increase may boost the production of hydronium ions of subcritical water, an additional increase in the temperature can degrade the amount of total protein [13,18].

### 2.3. Color and pH of the Hydrolysates

The color and pH of *A. pectinata* hydrolysates are presented in Table 1. The color is an important parameter in the selection of food by consumers. Lightness (L*) values of the hydrolysates obtained in the various treatments ranged from 10.44 ± 0.06 to 22.93 ± 0.72, which was influenced by the presence of pigments in the raw materials as well as the release of the different compounds during the hydrolysis reaction [18,19,20]. Two important chroma values, a* (red–green) and b* (yellow to blue), ranged between −1.39 ± 0.18 and 3.47 ± 0.03 and 5.16 ± 0.05 and11.63 ± 0.10, respectively. However, among the different temperature treatments of hydrolysates, 200 °C showed the lowest lightness and the highest redness values, indicating that this temperature zone is suitable to extract the bioactive compounds from *A. pectinata* viscera. Hao et al. [7] also reported that the temperature range of 170–230 °C showed reliable results in extracting antioxidant compounds from abalone viscera during subcritical water extraction. Oyster hydrolysates obtained at 200 °C showed higher redness values according to previous studies [18].

The pH values of different hydrolysates showed a continuously increasing pattern, with temperature ranging from 5.7 to 9.5. Lower pH values at a lower extraction temperature indicated the release of different acidic compounds, such as organic acids, that are derivatives of sugars. With the increase in temperature, organic acids or other acidic compounds decomposed into other substances, such as salts [18,19].

### 2.4. Total Proteins, Total Sugars, and Maillard Reaction Products (MRPs) of Hydrolysates

**Figure 2:**
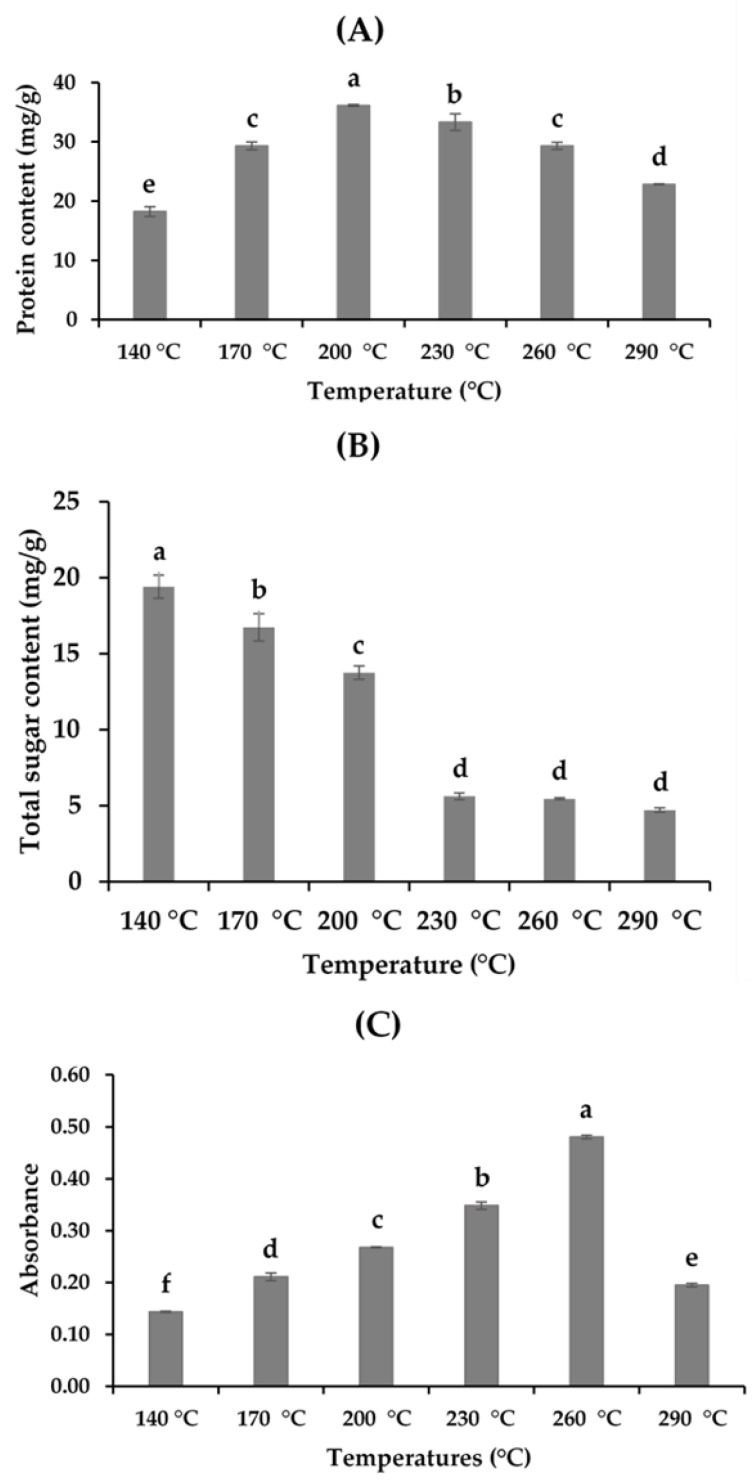
Physicochemical properties of the *A. pectinata* hydrolysates: (**A**) total protein (BSA mg/g); (**B**) total sugar content (mg/g); (**C**) Maillard reaction products (MRPs) (means with different superscripts differ significantly; *p* < 0.05).

Different compounds, such as total protein, total sugar, and the MRP content of the hydrolysates, in different extraction procedures are presented in Figure 2. Subcritical water extraction is a potential method to extract protein and peptide compounds. The amount of total protein varied from 18.24 ± 0.83 to 36.14 ± 0.16 mg/g BSA in the hydrolysates (Figure 2A). The crude protein content in the hydrolysates increased significantly with the increase in temperature from 140 to 200 °C. However, an additional increase in temperature showed a negative effect on proteins, with a continuous decline in the hydrolysates obtained. The possible cause for this extraction behavior might be related to the thermodynamics of the subcritical microenvironment of water. Temperature plays a significant role in the subcritical microenvironment, influencing the polarity of subcritical water by producing H^+^ and OH^−^, which is directly related to the dissolving capacity of polar organic compounds, such as protein [5,8,19]. Although an elevated temperature has a positive effect on the extraction of protein molecules by decreasing the dielectric constant of water, a high temperature also enhances the degradation of protein [15].

The amount of total sugars in the hydrolysates varied from 19.14 ± 0.76 4.71 ± 0.14 mg/g (Figure 2B) at various extraction temperatures. Hydrolysates obtained at 140 °C showed the highest sugar content, comparing all the hydrolysates, and the total amount of crude sugars decreased with the increase in the extraction temperature. The results obtained indicated that high temperatures enhance the conversion of sugars to different organic acids, which affects the sugar content of the hydrolysates. A similar pattern of results was also obtained by Hao et al. [7] for abalone viscera, where the total sugar content decreased from 17.9% to 0.8% at a temperature range of 140–230 °C during SWH. The intensity of the MRPs was measured at 420 nm, and the results ranged between 0.14 and 0.48 (Figure 2C). The release of MRPs increased with temperature, and the maximum value was obtained at 260 °C, whereas the absorbance reduced at 290 °C, which could be attributed to the degradation of different proteins at high temperatures [18,19].

### 2.5. Molecular Size of the Protein

**Figure 3:**
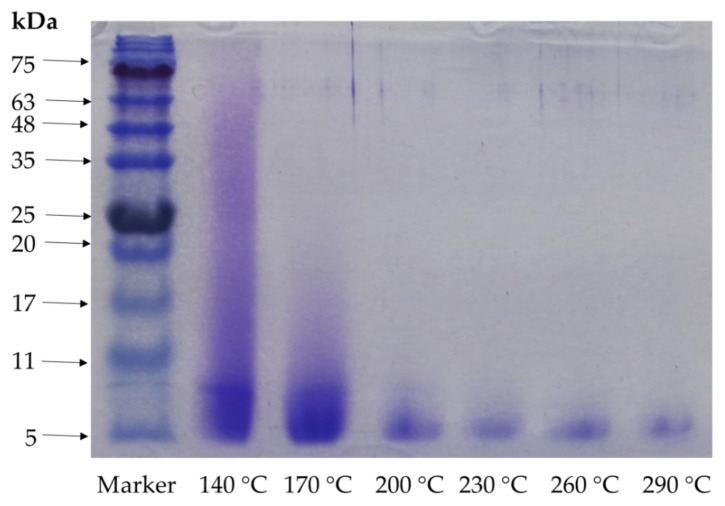
Results of SDS-PAGE analysis of *A. pectinata* hydrolysates at different temperatures (different arrow on the figure indicating the molecular weights of peptides).

The molecular weight of the protein present in the hydrolysates is depicted in the SDS-PAGE in Figure 3, which clearly indicated that a higher temperature reduced the molecular weights. The hydrolysate peptides obtained at 140 and 170 °C showed 75 and 17 kDa molecular weights, respectively, whereas 200 °C showed 11 kDa, and all the hydrolysates higher these temperatures showed 5 kDa as the highest molecular weight. A subcritical water microenvironment is highly influenced by the temperature, which directly affects the molecular size of the peptides [15]. Our study results showed that a higher temperature boosted the degradation of the protein obtained from comb penshell viscera using subcritical water extraction that can be used for oligopeptide production. The molecular weight profile of peptides is an important property that influences functional activity [13].

### 2.6. Amino Acid Composition

The amino acid composition of the hydrolysates is presented in Table 2. A total of eight essential amino acids (172 mg/g sample) were present in the raw sample; among them, leucine was the most dominant one. During the hydrolysis process, leucine, lysine, and valine showed the highest extraction efficiency until the temperature of 260 °C, whereas another amino acid, threonine, was not detected at the temperature of 230 °C or above. Threonine is a thermosensitive essential amino acid that was also undetected at 230 °C in a previous study [7]. The amount of essential amino acids decreased significantly at 290 °C, which might have been caused by the degradation effect at a high temperature. The taurine content was the highest in all *A. pectinata* hydrolysates, regardless of the extraction temperature. A total of 74.80 mg/g amino acid was obtained at 230 °C, combining both essential and non-essential amino acids. A temperature range of 170–230 °C proved to be the best temperature range to extract the protein and amino acid using subcritical water temperature. Previous studies on peptide extraction from abalone viscera using subcritical water have also found a high extraction efficiency at a temperature of about 200 °C [7].

### 2.7. Antioxidant Activities

**Figure 4:**
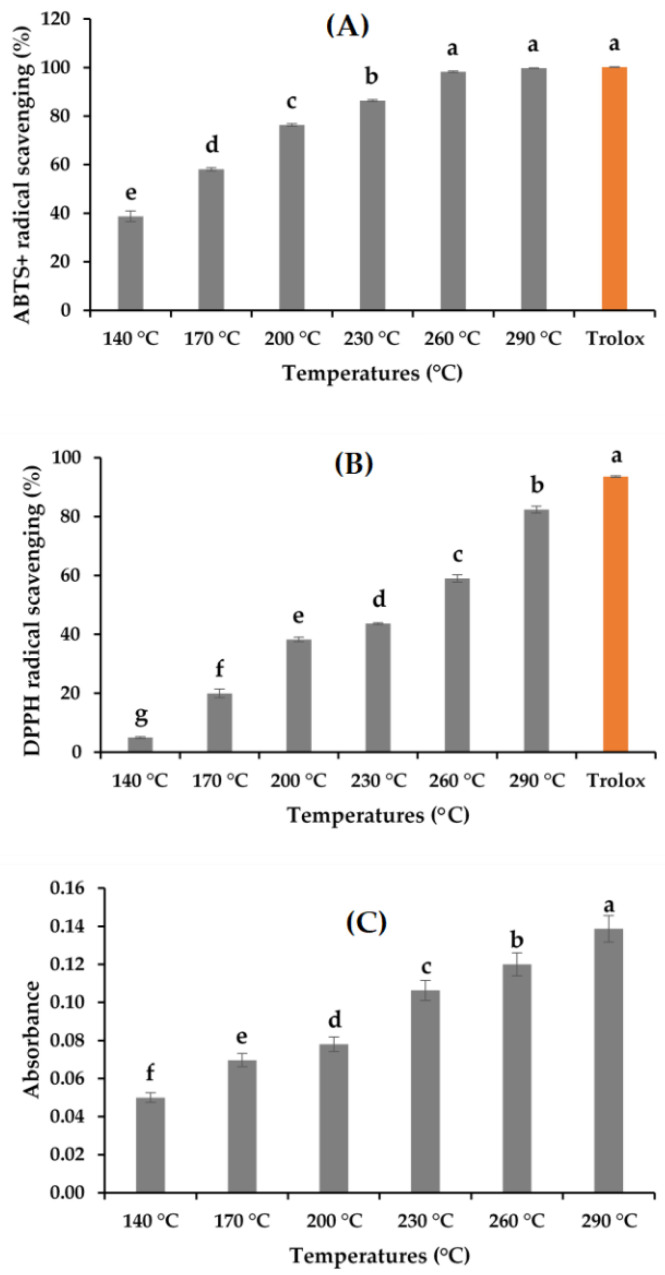
Antioxidant activities of the *A. pectinata* hydrolysates: (**A**) ABTS^+^ RSA; (**B**) DPPH RSA; (**C**) FRAP activity (means with different superscripts differ significantly; *p* < 0.05).

The antioxidant activities of the *A. pectinata* hydrolysates determined following the ABTS^+^, DPPH, RSA, and FRAP assay are depicted in Figure 4A–C. The ABTS^+^ RSA of the hydrolysates ranged from 38.71% ± 2.22% to 99.73%, and DPPH activity ranged from 4.95% ± 0.34% to 82.42% ± 1.12%. The RSA increased with the increase in the hydrolysis temperature. The trends of the increasing patterns of both ABTS^+^ and DPPH RSA can be correlated with the MRPs produced in SWH [13,18,19]. The production of small bioactive peptides increased with the hydrolysis temperature [13]. The hydrolysates obtained showed a greater ABTS^+^ RSA compared with the DPPH activity. The possible reason behind the mechanism of these two radical scavenging assays is that ABTS^+^ radical scavenging is more applicable in hydrophilic and lipophilic substances, whereas DPPH activity is much more influenced by the hydrophobic system [21]. FRAP activity was expressed by observing the absorbance value, which also showed a trend similar to ABTS^+^ and DPPH activities. Although the high temperature of the subcritical environment showed better antioxidant activities, the possible organic degradation of the targeted compounds must be considered during the subcritical water extraction process.

### 2.8. Antihypertensive Activity

**Figure 5:**
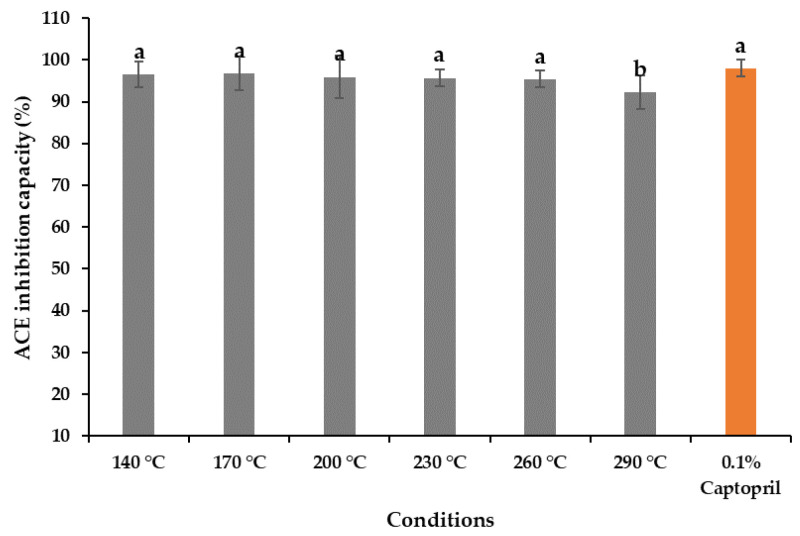
Antihypertensive activity of the *A. pectinata* hydrolysates obtained at various temperatures with standard (means with different superscripts differ significantly; *p* < 0.05).

The antihypertensive activity of the *A. pectinata* hydrolysates ranged from 96.77% ± 0.14% to 92.16% ± 0.04%, compared with 0.1% captopril (97.97% ± 0.68%), depicted in Figure 5. The hydrolysates obtained at 170 °C showed the highest ACE-inhibitory effects, whereas the lowest value was obtained at 290 °C, which is significantly lower than that of the other hydrolysates. The amino acid analysis presented in Table 2 also supports this behavior, indicating that the degradation of peptides and amino acids occurs at such a high temperature. The preparation of bioactive peptides during the subcritical hydrolysis might be the reason for the antihypertensive activity shown in hydrolysates [19]. Several studies have reported that the peptides obtained from marine bivalve hydrolysates contain antihypertensive properties [19,22].

### 2.9. Blood Clotting Activity

**Figure 6:**
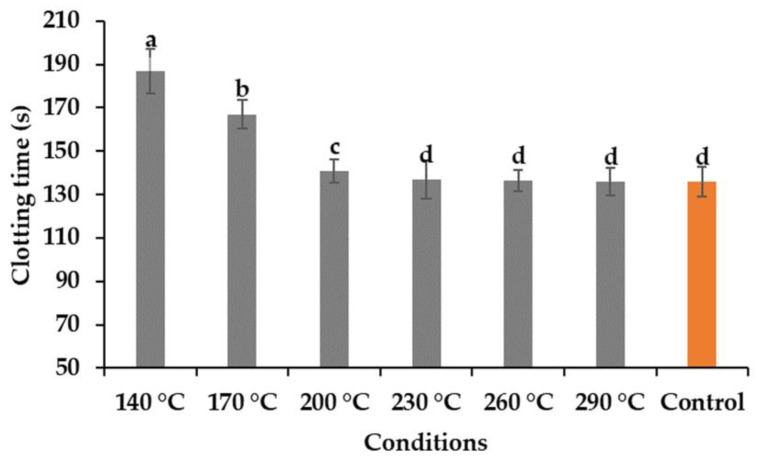
Anticoagulating activity of the hydrolysates at different temperatures (means with different superscripts differ significantly; *p* < 0.05).

The aPTT test was conducted to analyze the anticoagulant activity of the hydrolysates obtained, and the results presented in Figure 6. Human plasma from healthy donors was used for this analysis, and DW was used as the control to compare the results. However, the results showed that the hydrolysates obtained at temperatures of 140 and 170 °C showed some effects on the anticoagulant activity on human blood plasma. Nasri et al. [23] reported that the anticoagulant activity of the fish protein hydrolysate is influenced largely by the presence of amino acid compositions, molecular weights, and the sequence of the peptides [14]. Ho et al. [24] reported that alginate hydrolysate obtained by treating subcritical water at a temperature range of 110 to 130 °C showed a better result compared to higher temperatures.

## 3. Materials and Methods

### 3.1. Sample Preparation and Chemicals

The whole comb pen shell (*A. pectinata*) was kindly provided by the Hanlyo Fishery company, Sancheon-city, Gyeonnam province, Republic of Korea, and transported to the laboratory in an ice box. The body parts of the bivalves were peeled properly and washed. Separated parts were freeze-dried at −80 °C using a freeze-drier (HyperCOOL, HC 8080, Gyrozen Co., Ltd., Daejeon, Korea). Dried samples were crushed using a mechanical blender and sieved through a 210 µm mesh. Sieved powders were stored in an airtight bottle and kept at −72 °C until further extraction. Nitrogen gas used to pressurize the SWH system was collected from KOSHEM (Yangsan, Korea). Standards and reagents used for the biological activity tests were supplied by Sigma-Aldrich, Co. (St. Louis, MI, USA). All chemicals used for this study were of high-performance liquid chromatography (HPLC) or analytical grade.

### 3.2. Proximate Composition Analysis

The proximate composition of the freeze-dried *A. pectinata* viscera was analyzed following our previous methods [19]. A moisture analyzer (MX-50, A&D Instruments Ltd., Oxfordshire, UK) was used to complete the drying of the sample at 105 °C for 24 h. A muffle furnace was used to determine the ash content at 600 °C for 6 h, whereas crude lipid was determined by the Soxhlet extraction method, and crude protein was analyzed by the Kjeldahl method.

### 3.3. Subcritical Water Hydrolysis

Hydrolysates were prepared following the method of our previous work [16], with modification. Briefly, lyophilized *A. pectinata* viscera powder (5 g) and distilled water (150 mL) were premixed properly and added to a 200 mL batch reactor (Phosentech Co., Ltd., Daejeon, Republic of Korea). The loaded reactor was closed, and an electric heater was used to obtain the required temperature. The extraction temperatures ranged from 140 to 290 °C, with an interval of 30°C, and pressure was kept constant to maintain water in the liquid state. The extraction time fixed at 15 min was counted when the temperature was raised to the target value. The hydrolysates obtained were filtered using F1091-110 filter paper (CHMLAB GROUP, Barcelona, Spain) and stored at −24°C until further analysis. Lyophilized hydrolysate powder was used for the amino acid analyses. The solid extract obtained by lyophilization was dried completely, and the hydrolysis efficiency (HE) was calculated using the following formula:

HE (%) = (*S_h_*/*S_r_*) × 100 (1)

where *S_r_* is the weight of the raw sample before the extraction and *S_h_* is the weight of the dried extract obtained after filtration.

### 3.4. Color and pH Measurements

A reflectance colorimeter (Lovibond RT series, Tintometer Ltd., Amesbury, UK) was used to measure the color of hydrolysates in three different parameters, L*, a*, and b*, following our previous work [16]. The pH was measured using a pH meter (ORION STAR A211, Thermo Fisher Scientific, Pittsburgh, PA, USA) at ambient temperature. The pH meter was calibrated using technical buffer solutions of pH 4.00, 7.00, and 9.00 before the measurement.

### 3.5. Maillard Reaction Product (MRP) Measurements

The MRPs of the hydrolysates obtained were determined using an absorbance of 420 nm in a microplate spectrophotometer (Synergy HT, BioTek Instruments, Winooski, VT, USA). *A. pectinata* hydrolysates were diluted 20 times using double distilled water, and spectrometric observations were measured immediately after dilution.

### 3.6. Total Sugar Measurements

Total sugar was measured following the methods of our previous study [25], with modifications. Briefly, 0.5 mL of diluted *A. pectinata* hydrolysate was mixed with 1 mL of 2% phenol and 2.5 mL of concentrated H_2_SO_4_ and kept in the dark for 10 min. Mixed samples were kept at 22 °C for 30 min, and then absorbance was measured. A standard curve (50–200 µg/mL) was prepared using glucose, fructose, and galactose at a 1:1:1 ratio at 490 nm absorbance.

### 3.7. Determination of Total Protein

The total protein present in the *A. pectinata* hydrolysate was determined using the Pomory assay [26]. Chemicals used for the total protein analysis were 1 N Folin-Ciocalteu reagent, 1% CuSO_4_.5H_2_O, 2% tartrate, and 2% Na_2_CO_3_, which were used to prepare the fresh solution at a mixing ratio of 1:1:100. The fresh solution (5 mL) was mixed with 0.5 mL of the hydrolysate. After 10 min of reaction, 0.5 mL of Folin-Ciocalteu reagent was added, and the mixture was kept for 2 h; the mixture was then measured at 660 nm absorbance. Bovine serum albumin (BSA) was used as a standard to express the amount of protein, and the result obtained was expressed as mg BSA per gram of dried sample (mg BSA/g).

### 3.8. In Vitro Antioxidant Activities

#### 3.8.1. ABTS^+^ Radical Scavenging Activity

The ABTS^+^ radical scavenging activity (RSA) was analyzed following the work of Haq et al. [27], with modifications. Briefly, a stock solution was prepared from freeze-dried *A. pectinata* hydrolysate extracts (4 mg/mL) and 100 µL of the stock solution mixed with previously prepared 0.9 mL ABTS^+^ solution adjusted at an absorbance of 1.24 ± 0.05 at 734 nm after 6 min of reaction at room temperature under dark conditions. A standard Trolox solution (10 µg/mL) was used for comparison using the same volume. The ABTS^+^ RSA obtained by the hydrolysates was expressed as a percentage and determined by the following equation:

ABTS^+^ RSA (%) = [1 − (A_1_ − A_2_)/A_3_] × 100 (2)

A_1_ is the absorbance of the extracts with ABTS^+^, A_2_ is the absorbance of the blank (extracts mixed with distilled water), and A_3_ is the absorbance of the ABTS^+^ solution at 734 nm.

#### 3.8.2. DPPH Radical Scavenging Activity

The DPPH RSA was analyzed following the method of Roy et al. [9], with little modification. Aliquots of 100 µL volumes from the prepared stock solution (4 mg/mL) were mixed with 0.9 mL of 0.2 mM DPPH solution and kept in a dark place for 30 min. After completion of the reaction, absorbance was measured at 517 nm. Trolox solution (10 µg/mL) was used as the standard for comparison. The DPPH RSA was also expressed as a percentage and determined by the following equation:

DPPH RSA (%) = [1 − (A_1_ − A_2_)/A_3_] × 100 (3)

A_1_ is the absorbance of the extracts with DPPH, A_2_ is the absorbance of the blank (extracts mixed with distilled water), and A_3_ is the absorbance of the DPPH solution at 517 nm.

#### 3.8.3. Ferric Reducing Antioxidant Power Assay (FRAP)

The FRAP was analyzed following the methods of Haq et al. [27], with modifications. A volume of 100 µL of stock solution (4 mg/mL) was mixed with previously prepared 2.5 mL potassium ferricyanide and subsequently mixed with 2.5 mL of 0.2 M sodium phosphate buffer (pH 6.6). The combined mixture was subjected to incubation for 20 min at 50 °C, and 2.5 mL of 10% trichloroacetic acid was added to the mixture after cooling. The mixture was vortexed, and 2.5 mL of the mixture was mixed with the same the volume of distilled water with 0.5 mL FeCl_3._ The absorbance of the solutions was measured at 700 nm.

### 3.9. Sodium Dodecyl Sulfate-Polyacrylamide Gel Electrophoresis (SDS-PAGE)

Molecular weight of protein was determined by SDS-PAGE using a Mini-PROTEAN Tetra cell (Bio-Rad, Hercules, CA, USA). In chemical preparation steps, a running buffer was prepared by dissolving Tris-base (1.565 g), glycine (7.2 g), and SDS (0.5 g) in 400 mL of double distilled water (DW), and then adjusted to 500 mL using DW. Loading buffer was prepared by mixing 5 mL of 10% SDS, 2.5 mL of 25% glycerol, 2 mL of 1 M Tris-base (pH 6.8), 0.10 mL of bromophenol blue (1%), and 0.5 mL of mercaptoethanol. Stacking gel (5%) containing 3.4 mL of DW, 0.83 mL of 30% acrylamide mix, 0.63 mL of 1 M Tris (pH 6.8), 0.05 mL of 10% SDS, 0.05 mL of 10% ammonium persulfate, and 0.005 mL of tetramethylethylenediamine (TEMED) was prepared. Separating gel (12%) was a mixture of DW (3.3 mL), 30% acrylamide mix (4 mL), 1.5 M Tris (pH 6.8) (2.5 mL), 10% SDS (0.1 mL), 10% ammonium persulfate (0.05 mL), and TEMED (0.004 mL). Staining buffer contained 1 g of Coomassie R250, 100 mL of acetic acid (glacial), 400 mL of methanol, and 500 mL of DW, whereas the de-staining buffer was prepared by mixing 200 mL of methanol, 100 mL of acetic acid (glacial), and 700 mL of DW. In the sample treatment, the samples (0.33 mL) were mixed with 0.5 mL of 0.02 M sodium phosphate buffer (pH 7.2), 0.5 mL of the loading buffer, and the resulting mixture was heated at 90°C for 5 min. The heated mixture was centrifuged for 5 min at 5500 rpm, and the supernatant was collected for analyses. 

The SDS-PAGE protocol is briefly described as follows. An appropriate amount of separating gel was filled into the gap between the glass plates which had been fixed in the casting frame. To make the gel horizontal, 1–2 mL of isopropanol was filled into the gap until overflow. After 20–40 min, the upper liquid was discarded, and the stacking gel was filled into the gap until overflow. Right after, a well-forming comb was carefully inserted. After completing the gelation process (20–30 min), the comb was carefully removed, and the glass plates were properly set in the cell buffer dam. Running buffer was poured into the inner chamber, and pouring was continued after overflow until the buffer surface reached the required level in the outer chamber. Amounts of 10 µL of the treated samples or protein marker (standard) were loaded into wells. The chamber was covered to connect the anodes, and an electricity power with a voltage of 120 V was applied to the system. The running time was approximately 2 h to complete the experiment. Next, the gel was stained in staining buffer for 1 h at room temperature and, consequently, destained in destaining buffer overnight at room temperature. The destaining buffer was changed several times when the color became dark. Finally, the obtained gel was carefully removed from the destaining reservoir, and the result was achieved.

### 3.10. Amino Acid Analysis

The presence of amino acids in the hydrolysate was determined following the methods reported in a previous study [28]. The sample (2.0 g) was taken in a beaker and mixed with 30 mL of 70% ethanol and subjected to ultra-sonication for 60 min. The sonicated sample was kept overnight at room temperature and filtered using a 0.2 m syringe filter (hydrophilic). The filtered samples were analyzed by HPLC (Thermo Fisher Scientific, Pittsburgh, PA, USA) equipped with a fluorescence detector (1260 FLC, Agilent, Santa Clara, CA, USA). A VDSpher 100 C18-E (4.6 mm × 150 mm, 3.5 µm; Optilab, Berlin Germany) was used for the sample separation, with 35 min of analysis time.

A small volume of the sample (0.5 µL) was injected into the HPLC system, where the column temperature was maintained at exactly 40 °C. Two mobile phases were used during analysis, where 40 mM sodium phosphate (pH 7.0) was used as mobile phase A, and a mixture of DW, acetonitrile, and methanol (10:45:45 ratio) was used as mobile phase B. An absorbance of 338 nm was used for the analyses of free amino acids. Total amino acids were extracted using 6 N NaOH instead of using 70% ethanol following the abovementioned procedure at 130 °C for 24 h. The obtained mixture was diluted with DW and filtered after neutralization. Total amino acids were also measured following the abovementioned HPLC protocol used for the determination of free amino acids.

### 3.11. Antihypertensive Analysis

The ACE kit-WST manual (Dojindo Molecular Technologies, Rockville, MD, USA) was followed to determine the antihypertensive activity of the extracts. Briefly, 1.0 mg/mL sample solution was prepared using HPLC grade water. Equal volumes of the substrate buffer and water were mixed with 1.0 mL of the prepared solution and incubated for 1 h at 37 °C. The mixtures were kept at room temperature and then measured at 450 nm absorbance. Two blank solutions were run following the abovementioned protocol using the substrate buffer (blank 1) and deionized water (blank 2) to determine ACE-inhibitory activity, and the results were compared with standard captopril (0.1%) solution. ACE-inhibitory activity was expressed as a percentage using the following equation:

ACE-inhibitory activity (%) = [(A_blank1_ − A_sample_)/(A_blank1_ − A_blank2_)] × 100 (4)

### 3.12. In Vitro Anticoagulant Activity

The in vitro anticoagulant activity of the extracts was measured according to the methods of Pawlaczyk et al. [29] and Ho et al. [30]. The concentration of the extract used for the experiments was 4.0 mg/mL. The activated partial thromboplastin time (aPTT) test was carried out in triplicate, and the PTT reagents were bought from Sigma-Aldrich Co. (St. Louis, USA).

### 3.13. Statistical Analyses

One-way analysis of variance (SPSS version 23, IBM, Chicago, IL, USA) was used for statistical analyses, after conducting the experiments in triplicate. The results obtained were expressed as the mean ± standard deviation. Values of *p* < 0.05 were considered significant, and significant differences of the mean were determined using Duncan’s multiple range test.

## 4. Conclusions

Hydrolysates obtained from *A. pectinata* viscera using SWH yielded a large amount of amino acids and showed other biopotential activities. The antioxidant activities of the hydrolysates increased with the increase in temperature of subcritical water. The highest amount of crude protein was obtained at 200 °C, but the sugar content reduced with the increase in the hydrolysis temperature. Our study revealed that the temperature range of 170–230 °C is suitable for the extraction of bioactive peptides, with a minimum degradation of proteins. All the hydrolysates showed exceptional hypertensive activities, whereas the hydrolysates obtained at 290 °C showed significantly lower activity, compared to other hydrolysates, which indicated the degradation of peptides to other biocomponents. Our raw material, *A. pectinata* viscera, proved to be an important source of peptides from marine biomass, with significant bioactivities. Moreover, SWH is an environmentally friendly method that can be used for the efficient valorization of *A. pectinata* viscera.

## Figures and Tables

**Table 1:** Color and pH of the *A. pectinata* hydrolysates obtained at various temperatures.

Conditions	L*	a*	b*	pH
140 °C	22.93 ± 0.72 ^a^	−1.39 ± 0.18 ^a^	5.16 ± 0.50 ^d^	5.68
170 °C	20.20 ± 0.12 ^a,b^	−1.19 ± 0.05 ^a^	9.11 ± 0.18 ^b^	5.83
200 °C	10.44 ± 0.06 ^d^	3.47 ± 0.03 ^e^	8.02 ± 0.030 ^c^	6.24
230 °C	19.7 ± 0.15 ^c^	1.47 ± 0.10 ^c^	11.16 ± 0.31 ^a^	8.36
260 °C	21.01 ± 0.04 ^b^	2.2 ± 0.01 ^d^	11.63 ±0.10 ^a^	8.92
290 °C	22.71 ± 0.14 ^a^	0.72 ± 0.01 ^b^	8.14 ± 0.09 ^c^	9.50

Means with different superscripts in the same row differ significantly (*p* < 0.05).

**Table 2:** Amino acid compositions in raw and hydrolyzed *A. pectinata* hydrolysates.

AMINO ACIDS	Raw Sample		Hydrolyzed Samples					
			140 °C	170 °C	200 °C	230 °C	260 °C	290 °C
	Total Amino Acids (mg/g)	Free Amino Acids (mg/g)	Free Amino Acids (mg/g)					
	**Essential amino acids (EAA)**							
Histidine	8.32	0.11	0.27	0.34	0.52	0.97	0.75	0.10
Isoleucine	21.4	0.24	0.50	0.59	0.89	1.22	1.38	ND
Leucine	32.31	0.36	0.87	0.94	1.72	2.70	2.89	0.44
Lysine	30.67	0.37	0.98	0.97	1.44	1.74	1.83	0.62
Methionine	12.58	0.10	0.42	0.62	1.00	1.35	0.61	ND
Phenylalanine	19.25	0.25	0.58	0.59	1.00	1.49	1.38	0.34
Threonine	24.27	0.36	0.49	0.47	0.54	ND	ND	ND
Valine	23.17	0.27	0.55	0.47	1.04	2.18	3.21	0.29
Total	172.00	2.06	4.66	4.99	8.15	11.70	12.10	1.79
	**Non-essential amino acids (NEAA)**							
Alanine	26.43	5.12	8.4	7.09	7.64	11.10	9.90	1.23
Arginine	35.88	2.08	3.35	2.65	2.46	1.40	0.09	0.10
Aspartic acid	26.43	1.45	2.50	4.08	3.03	1.25	0.38	0.10
Glutamic acid	75.53	3.84	4.78	0.27	0.17	0.19	0.20	0.09
Glycine	34.33	1.30	2.20	2.49	4.24	7.43	8.22	0.84
Proline	21.63	0.16	0.39	0.62	1.66	2.70	1.11	ND
Serine	23.17	0.66	1.46	1.55	2.30	0.87	0.07	0.07
Taurine	57.95	28.7	34.08	40.76	34.20	36.10	35.00	10.70
Tyrosine	19.20	0.4	0.76	0.79	1.49	2.13	1.73	0.24
Total	320.60	43.8	57.92	60.30	57.20	63.10	56.70	13.30
EAA + NEAA	492.52	45.8	62.58	65.29	65.30	74.80	68.80	15.10

ND = not detected.

## Data Availability

Not applicable.

## Supplementary Materials

The following are available online at https://www.mdpi.com/1660-3397/19/3/137/s1, Table S1. Proximate composition of lyophilized *Atrina pectinata* powder; Figure S1. Schematic diagram of laboratory scale subcritical water hydrolysis system used in this experiment; Figure S2. Chromatogram of total amino acids from the raw *A. pectinata* powder; Figure S3. Chromatogram of free amino acids from the raw *A. pectinata* powder; Figure S4. Chromatogram of amino acids obtained *from A. pectinata* powder at different subcritical extraction temperature.
