# Supplementary material for: Amino Acid Profiles and Biopotentiality of Hydrolysates Obtained from Comb Penshell (Atrina pectinata) Viscera Using Subcritical Water Hydrolysis
Source: Mar Drugs. 2021 Mar 1;19(3):137. doi: 10.3390/md19030137 (PMC7999596; doi:10.3390/md19030137)
Supplement: Supplementary file 1 [file marinedrugs-19-00137-s001.pdf]

Amino acid profiles and bio-potentiality of hydrolysates obtained from comb pen shell (*Atrina pectinata*) viscera using subcritical water hydrolysis

Hee-Jeong Lee, Vikash Chandra Roy, Truc Cong Ho, Jin-Seok Park, Yu-Rin Jeong, Seung-Chan Lee, Sung-Yeol Kim, Byung-Soo Chun*

Supplementary file

Table S1. Proximate composition of lyophilized *Atrina pectinata* powder

composition	Content (%)
	A. pectinata
Crude lipid	9.60 ± 0.13
Crude protein	60.70 ± 0.11
Moisture	2.50 ± 0.02
Ash	17.20 ± 0.05
Carbohydrate	9.98 ± 0.05

Values are expressed as mean ± SD.

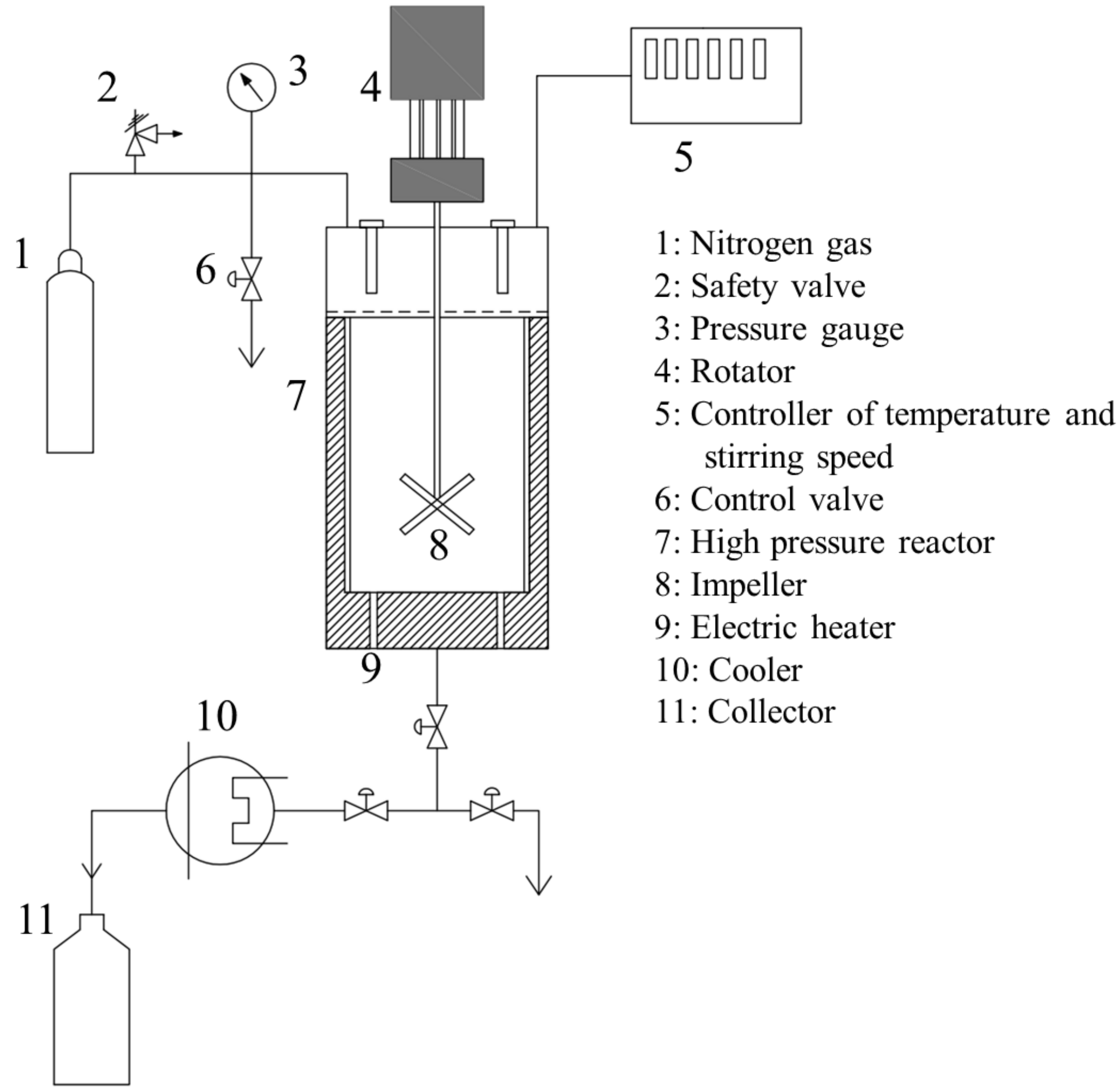

Figure S1. Schematic diagram of laboratory scale subcritical water hydrolysis system used in this experiment

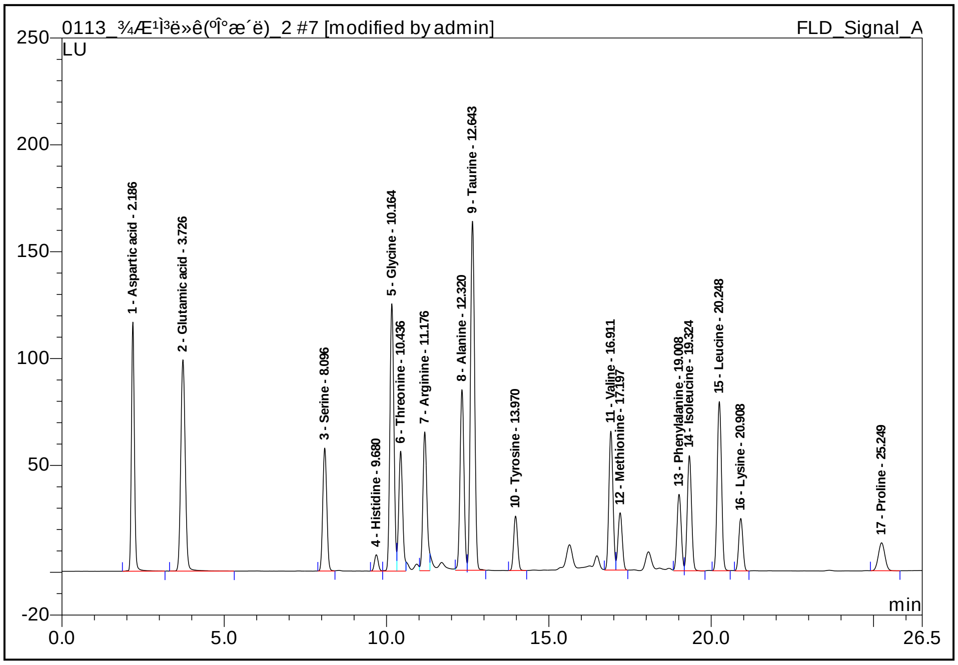

Figure S2. Chromatogram of total amino acids from the raw *A. pectinata* powder

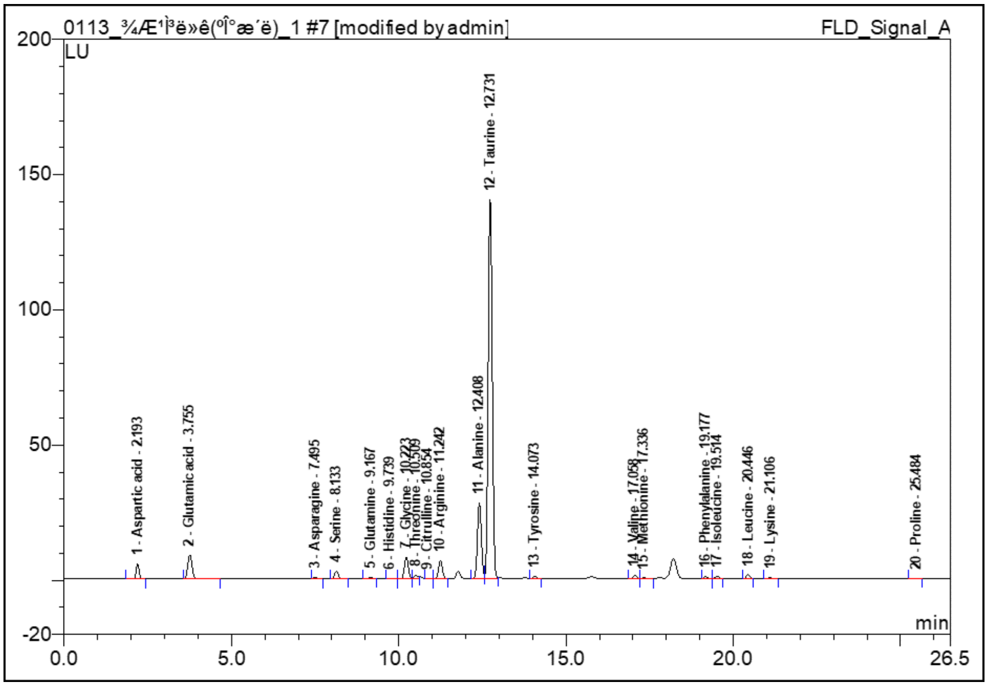

Figure S3. Chromatogram of free amino acids from the raw *A. pectinata* powder

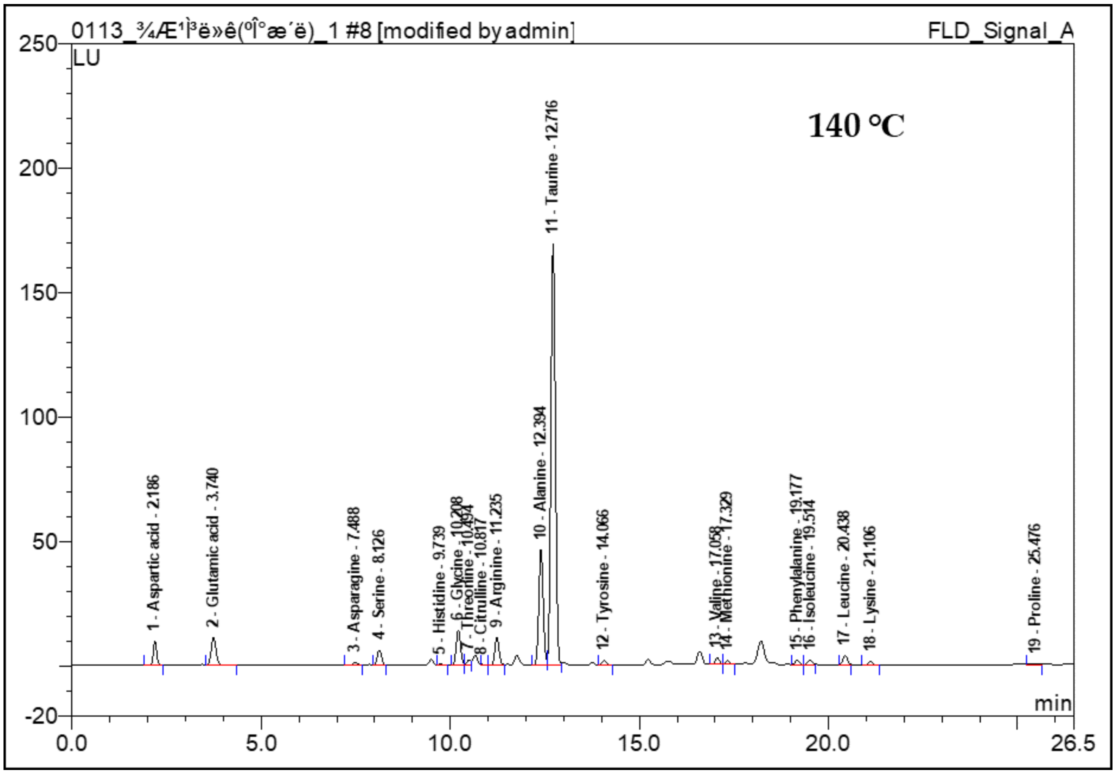

[modified by admin]
FLD_Signal_A
LU
140 °C
1 - Aspartic acid - 2.186
2 - Glutamic acid - 3.740
3 - Asparagine - 7.488
4 - Serine - 8.126
5 - Histidine - 9.739
6 - Glycine - 10.208
7 - Threonine - 10.494
8 - Citrulline - 10.817
9 - Arginine - 11.235
10 - Alanine - 12.394
11 - Taurine - 12.716
12 - Tyrosine - 14.066
13 - Valine - 17.058
14 - Methionine - 17.329
15 - Phenylalanine - 19.177
16 - Isoleucine - 19.514
17 - Leucine - 20.438
18 - Lysine - 21.106
19 - Proline - 25.476
min

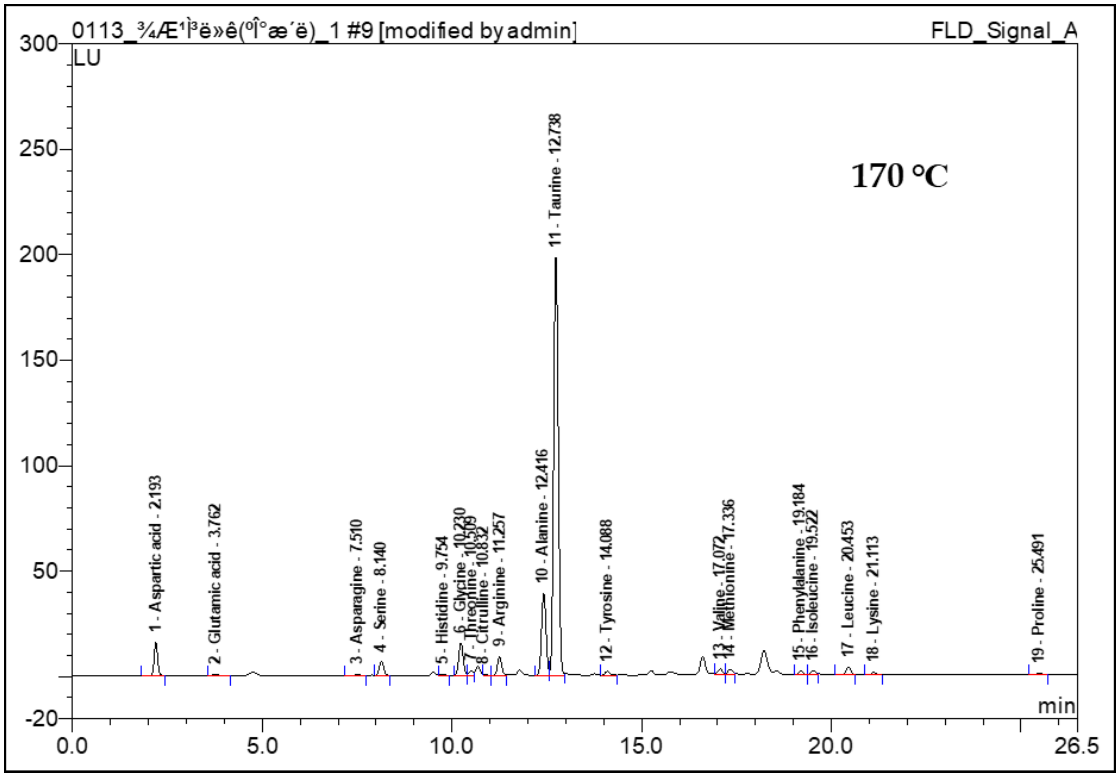

[modified by admin]
FLD_Signal_A
LU
170 °C
1 - Aspartic acid - 2.193
2 - Glutamic acid - 3.762
3 - Asparagine - 7.510
4 - Serine - 8.140
5 - Histidine - 9.754
6 - Glycine - 10.230
7 - Threonine - 10.509
8 - Citrulline - 10.832
9 - Arginine - 11.257
10 - Alanine - 12.416
11 - Taurine - 12.738
12 - Tyrosine - 14.088
13 - Valine - 17.072
14 - Methionine - 17.336
15 - Phenylalanine - 19.184
16 - Isoleucine - 19.522
17 - Leucine - 20.453
18 - Lysine - 21.113
19 - Proline - 25.491
min

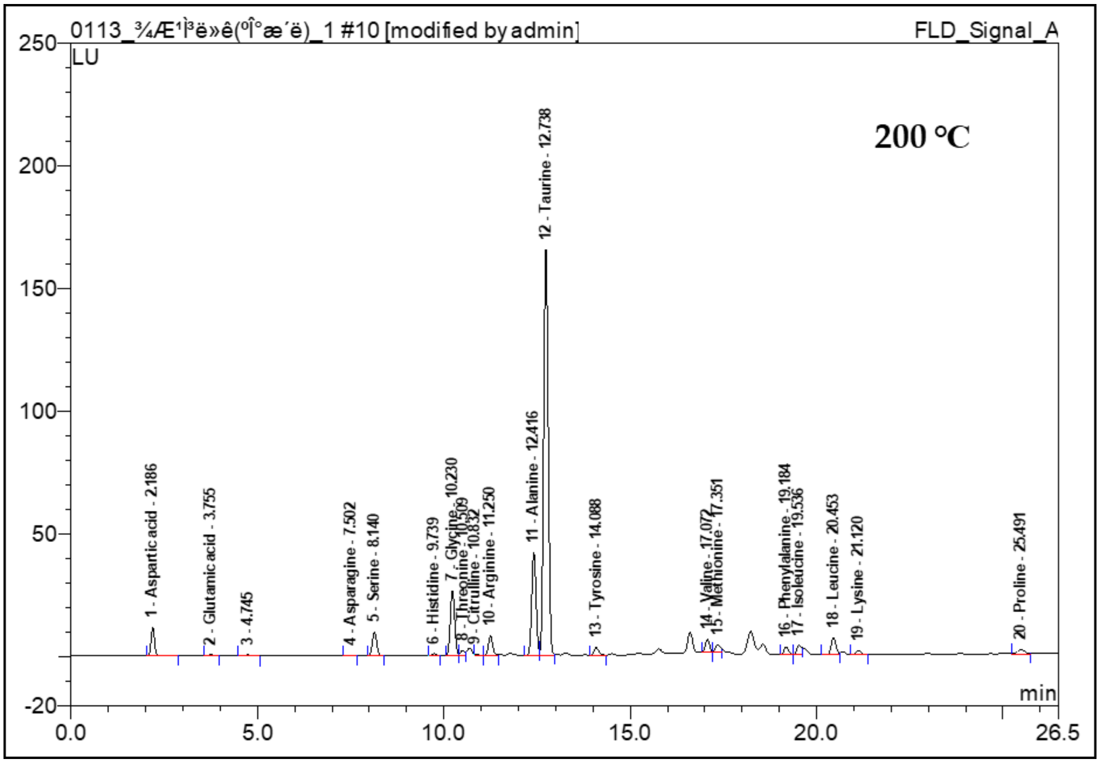
0113_¾Æ¹Ì³ë»ê(º¹°æ´ë)_1 #10 [modified by admin]
FLD_Signal_A
LU
200 °C
1 - Aspartic acid - 2.186
2 - Glutamic acid - 3.755
3 - 4.745
4 - Asparagine - 7.502
5 - Serine - 8.140
6 - Histidine - 9.739
7 - Glycine - 10.230
8 - Threonine - 10.509
9 - Citrulline - 10.832
10 - Arginine - 11.250
11 - Alanine - 12.416
12 - Taurine - 12.738
13 - Tyrosine - 14.088
14 - Valine - 17.072
15 - Methionine - 17.351
16 - Phenylalanine - 19.184
17 - Isoleucine - 19.536
18 - Leucine - 20.453
19 - Lysine - 21.120
20 - Proline - 25.491
min
250
200
150
100
50
-20
0.0
5.0
10.0
15.0
20.0
26.5

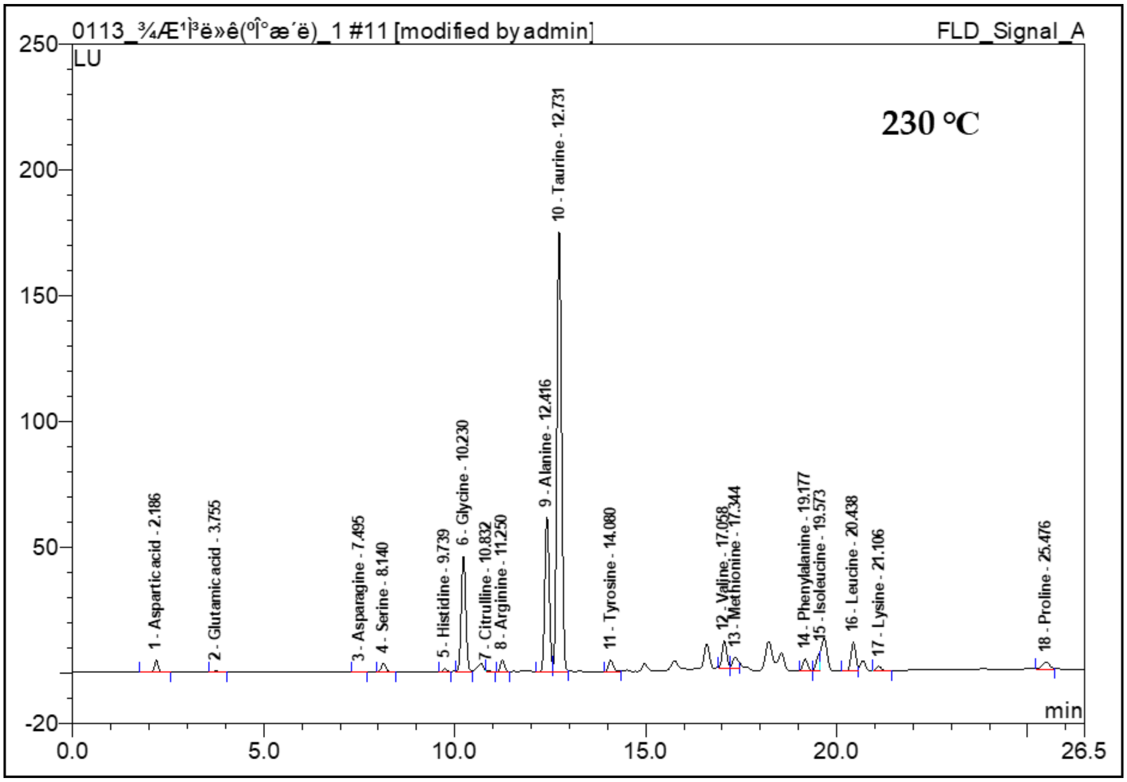
0113_¾Æ¹Ì³ë»ê(º¹°æ´ë)_1 #11 [modified by admin]
FLD_Signal_A
LU
230 °C
1 - Aspartic acid - 2.186
2 - Glutamic acid - 3.755
3 - Asparagine - 7.495
4 - Serine - 8.140
5 - Histidine - 9.739
6 - Glycine - 10.230
7 - Citrulline - 10.832
8 - Arginine - 11.250
9 - Alanine - 12.416
10 - Taurine - 12.731
11 - Tyrosine - 14.080
12 - Valine - 17.058
13 - Methionine - 17.344
14 - Phenylalanine - 19.177
15 - Isoleucine - 19.573
16 - Leucine - 20.438
17 - Lysine - 21.106
18 - Proline - 25.476
min
250
200
150
100
50
-20
0.0
5.0
10.0
15.0
20.0
26.5

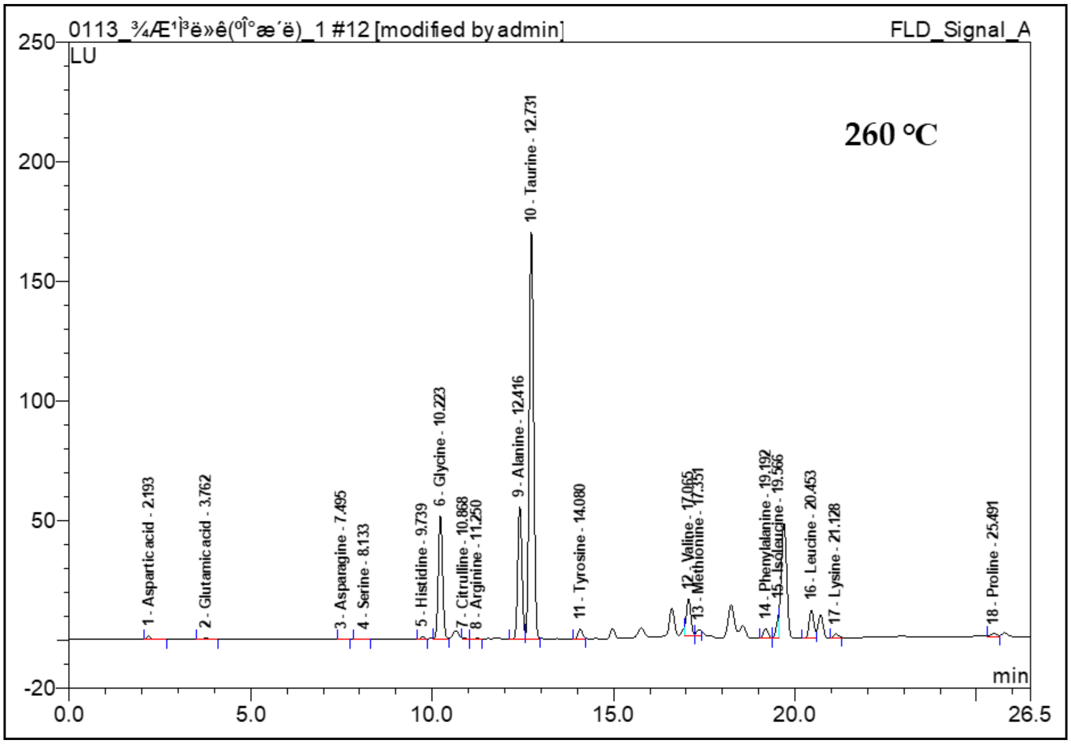

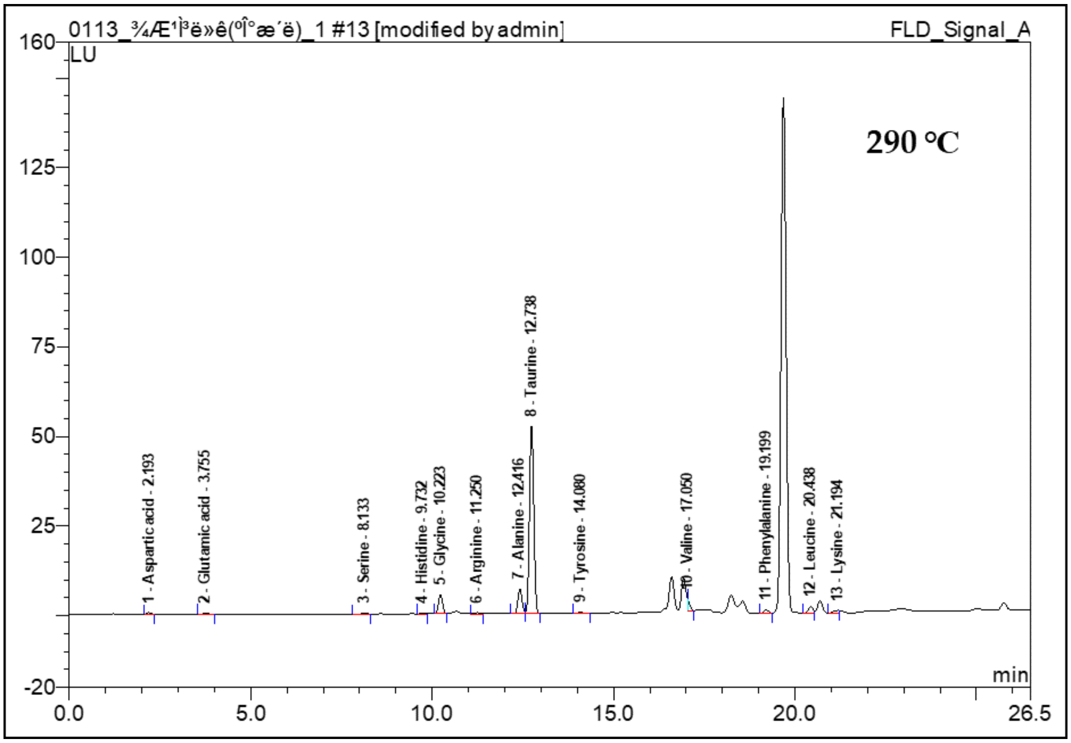

Figure S4. Chromatogram of amino acids obtained from *A. pectinata* powder at different subcritical extraction temperature.
